# The Role of Alpha-Synuclein Deposits in Parkinson’s Disease: A Focus on the Human Retina

**DOI:** 10.3390/ijms24054391

**Published:** 2023-02-23

**Authors:** Mariachiara Di Pippo, Serena Fragiotta, Federico Di Staso, Luca Scuderi, Solmaz Abdolrahimzadeh

**Affiliations:** 1 Ophthalmology Unit, Neurosciences, Mental Health, and Sensory Organs (NESMOS) Department, St. Andrea Hospital, Sapienza University of Rome, Via di Grottarossa 1035/1039, 00189 Rome, Italy; 2 Connecticut Uveitis Foundation, 1043 Farmington Avenue, West Hartford, CT 06107, USA; 3 Department of Sense Organs, Sapienza University of Rome, 00161 Rome, Italy

**Keywords:** Parkinson’s disease, spectral domain optical coherence tomography, retina, α-synuclein, Lewy bodies, retinal imaging, dopamine, retinal amacrine cells

## Abstract

Parkinson’s disease (PD) is a neurodegenerative condition characterized by the progressive deterioration of dopaminergic neurons in the central and peripheral autonomous system and the intraneuronal cytoplasmic accumulation of misfolded α-synuclein. The clinical features are the classic triad of tremor, rigidity, and bradykinesia and a set of non-motor symptoms, including visual deficits. The latter seems to arise years before the onset of motor symptoms and reflects the course of brain disease. The retina, by virtue of its similarity to brain tissue, is an excellent site for the analysis of the known histopathological changes of PD that occur in the brain. Numerous studies conducted on animal and human models of PD have shown the presence of α-synuclein in retinal tissue. Spectral-domain optical coherence tomography (SD-OCT) could be a technique that enables the study of these retinal alterations in vivo. The objective of this review is to describe recent evidence on the accumulation of native or modified α-synuclein in the human retina of patients with PD and its effects on the retinal tissue evaluated through SD-OCT.

## 1. Introduction

Parkinson’s disease (PD) is a widespread neurodegenerative condition affecting between 7 and 10 million people worldwide [1]. Classic neuropathological PD hallmarks are the progressive degeneration of dopaminergic neurons of the substantia nigra in the midbrain that cause reduced levels of striatal dopamine levels, accumulation of improperly folded cytoplasmatic α-synuclein (α-syn)—an intraneuronal protein present in Lewy bodies (LBs), and Lewy neuritis (LNs) [2].

α-syn is a neuronal protein of 140 amino acids expressed in large quantities in the presynaptic terminals of midbrain dopaminergic neurons, where it regulates nigrostriatal neurotransmission and tyrosine hydroxylase (TH) activity. Its physiological functions remain unclear, but it has been hypothesized that it plays a role in synaptic function, maintenance, and plasticity of the central nervous system [3]. PD can be considered a multifactorial disease, where environmental and genetic factors determine the development of the condition. Among the environmental risk factors there are age, ethnicity (Hispanics have a higher risk of developing the disease), and exposure to pesticides. The contribution of genetics to PD is suggested by the increased risk of disease associated with a family history of PD or tremors. Monogenic forms of PD have been identified, including mutations in LRRK2 and Parkin, the most common causes of dominantly and recessively inherited forms, respectively. From a molecular point of view, the genetic and environmental factors trigger the hyperexpression of a-sin, leading to monomer accumulation, misfolding, and the absence of efficient clearance. The alteration of the metabolism of α-syn, observed both in the acquired forms and familiar forms of PD is believed to be due to the alteration of the genes involved in its correct transcription (such as PARK1) and in its elimination (such as PARK2 and PARK3, encoding for ubiquitin). Indeed, in affected patients the loss of molecular mechanisms that regulate protein homeostasis (chaperone inefficiency, mitochondrial dysfunction, presence of ROS, intracellular protein, and membrane trafficking impairment, etc.) has been observed. For example there is an abnormal expression of α-syn, with single amino acid replacement, such as Ala53Thr, Ala30Pro, leading to its major aggregation propensity, or its phosphorylation, such as that observed at Serine 129 (Ser 129), leading to misfolding of the protein [4]. This last modification is one of the most frequent alterations of α-syn and results in the formation of protofibrils that accumulate in the intracellular cytoplasm, causing the formation of LBs. These accumulations affect the proper metabolism of dopamine (DA) and its storage within vesicles resulting in the intracellular production of reactive oxygen species and neuronal death [5].

The histopathologic alterations are not confined to the substantia nigra but can be observed in the cortex, amygdala, locus coeruleus, vagal nucleus, and the peripheral autonomic nervous system leading to the appearance of the typical symptoms [6,7]. PD, from a clinical point of view, is characterized by the classic triad of tremor, rigidity, and bradykinesia as well as a set of non-motor symptoms and systemic autonomic dysfunctions linked to peripheral dopaminergic depletion [8,9]. The latter include pain, incontinence, constipation, sleep disorders, fatigue, anxiety, depression, and visual symptoms, that may emerge years or even decades before motor symptoms, the study of which could be of crucial importance for making an early diagnosis of disease [10,11].

Visual symptoms are present in almost 80% of PD patients and include deficits of visual acuity, spatial contrast sensitivity, and color vision, reported to correlate with disease progression [12,13,14]. Nevertheless, the biological mechanisms resulting in vision impairment in PD are not clear and the consequences of misfolded α-syn in the retina are not completely understood.

It is now known that the clinical signs of PD are evident when about 80% of striatal dopamine and 50% of nigral neurons are lost [15]. The importance of an early diagnosis is, therefore, fundamental in order to identify the disease in the pre-clinical phase and set up therapy as soon as possible. The retina may be the answer to this need as it can be considered “a window on the brain” for its similarities with brain tissue and its easy accessibility [16].

ß-amyloid accumulation and macro- and microscopic vascular changes are localized also to the retina, similar to the brain, in other neurodegenerative diseases such as Alzheimer’s disease, and stroke. This also applies to PD. Visual symptoms are usually present before the onset of motor symptoms and reflect the course of brain disease. Whether visual symptoms depend on retinal or central alterations, has yet to be clarified [17].

Physiologically, DA in the retina modulates light adaptation. Light activates DA release from amacrine cells, within the nuclear layer, and extracellular dopamine acts on D1-like dopaminergic receptors on amacrine, horizontal, and bipolar cells, and on D4-receptors on rod and cone cells. This induces amplification of the cone pathway producing a shift from rod-dominant to cone-dominant vision during daylight and implementing color vision. These visual functions are frequently compromised in Parkinsonian patients, although they respond positively to DA replacement therapy with levodopa [18,19].

Microscopic changes in the retinal tissue could result in structural/morphologic changes visible through modern retinal imaging techniques. Among these, spectral domain optical coherence tomography (SD-OCT) is certainly the most common method. SD-OCT is a non-invasive imaging technique that provides high-resolution retinal and choroidal scans, enabling an accurate study of the qualitative and quantitative features of the inner and external retinal layers and the choroid. This method is routinely used in the field of ophthalmology in pathologies such as age-related macular degeneration and diabetic retinopathy [20,21] and in rare retinal diseases with a neurological component [22,23,24]. If α-syn accumulates in the retina from the early stages of PD and is possibly detected with imaging techniques, then SD-OCT could be used to identify potential biomarkers for the early diagnosis of PD.

The objective of this review is to describe recent evidence on the accumulation of native or modified α-syn in the retina of patients with PD and its effects on retinal structure, evaluated through SDOCT. For this purpose, the literature, accessed through September 2022, was analyzed using the following keywords: “((α-synuclein) OR (alfa-synuclein) OR (synuclein) OR (Lewy bodies)) AND (retina) AND (Parkinson)” and “(Parkinson’s disease) AND (spectral domain optical coherence tomography)”. Regarding SD-OCT, articles from 2018 onwards were selected.

## 2. Alpha-Synuclein in Human Retina

Numerous studies have been conducted on the retinas of animal models and PD patients. Research has focused on the retinal regions of α-syn accumulation, the shape of the protein, if native or phosphorylated, and, where possible, the resulting physiopathological consequences. One of the earliest pieces of evidence is that of Martinez-Navarrete et al., who studied the expression of α-syn and its distribution pattern in vertebrates. The authors analyzed retinal sections using specific anti-α-syn antibodies in their native form. They observed accumulation of α-syn predominantly localized in the outer segments of the photoreceptors and axonal terminals in the outer plexiform layer (OPL), and in amacrine and bipolar cells in the inner plexiform layer (IPL). These results are partially in contrast to the report by Surguchov et al., who had previously described the accumulation of α-syn in the IPL but not in the OPL [25]. Martinez-Navarrete et al. hypothesized the involvement of α-syn in the neurotransmission between photoreceptors and bipolar and amacrine cells, and in the synaptic-vesicular cycle (storage of the neurotransmitter, its release, and reuse), similar to what happens in the brain [3].

Based on these observations, Leger et al., conducted a study on protein accumulation in the aging retina. The accumulation of α-syn was found in the intracytoplasmic spaces of the inner nuclear layer (INL) and the more advanced the age, the more the inclusions were numerous. Interestingly, the authors found a positive correlation between intracytoplasmic α-syn inclusions and ubiquitin inclusions. Ubiquitin is a regulatory protein responsible for “ubiquitinating” other proteins and thus determining their degradation at cellular level, and it is already known that this is one of the most represented biochemical components of LBs together with α-syn [26]. In aging, as well as in PD, there is an accumulation of intraretinal α-syn and ubiquitin, with a mechanism probably very similar to what occurs in the brain [3].

These findings opened the way to more in-depth studies on the retinal pathogenetic aspects that take place during PD. Beach et al. conducted research on the Serin-129 phosphorylated form of α-syn (p-syn) on human subjects with PD. In particular, the authors enrolled nine patients with a neuropathological diagnosis of PD, four with Lewy Body dementia, and 4 elderly healthy subjects. After preparing retinal whole-mounts, they immunohistochemically stained these with an antibody against α-syn phosphorylated at serine 129. In the patients with PD and three out of four with Lewy Body dementia, the α-synuclein-immunoreactive phosphorylated nerve fibers (p-syn IR) were located within the nerve fiber layer (NFL), the ganglion cell layer (GCL), and the inner layers of the IPL [27]. This is in contrast with the report of Ho et al., who did not find a pathological form of neuritis of α-syn in patients with PD, but only a diffuse intracytoplasmic immunoreactivity in the context of the GCL and the INL [28]. Beach et al. justified this different outcome to the methodological differences adopted in the two studies. Indeed, Beach et al. used larger retinal samples than those used by Ho et al. (whole-mount retinal versus retinal cross-sections) and used the anti-phosphorylated form of α-syn antibodies against the anti-unmodified form of α-syn antibodies. The p-syn nerve fibers detected in the study of Beach et al. were scattered in the retinal field [27]. The results of Beach et al. were later confirmed in a study by Mammadova et al. where retinal damage from the accumulation of the phosphorylated form of p-syn on mouse models of A53T-mutation PD form was detected [29].

Bodis-Wollner et al., in a study of 4 post-mortem eyes of PD patients and 12 healthy controls, described aggregates of α-syn within neurons in the GCL, INL, and IPL for the first time in 2014. In particular, the authors observed intraneuronal globular LBs in the INL, LNs, and diffuse α-syn in the IPL, and intra- and extra-cellular inclusions of α-syn in the GCL. Moreover, the cells in which intracellular accumulates of α-syn were evident presented straddling between the INL and IPL, had the morphological and distribution characteristics of the retinal dopaminergic amacrine cells. Based on this observation, the authors tried to double-stain these cells for tyrosine hydroxylase but without success. This result is in line with the literature where the more advanced the damage with greater reactivity for α-syn, the lower the immunoreactivity for tyrosine-hydroxylase [30]. Another very interesting point underlined by the authors was the presence of inclusions of α-syn at GCL level. This could be linked either to an overexpression of the α-syn gene or to the propagation of α-syn from neurons of the INL and IPL to GCL cells. However, there are no conventional synapses between these two cell populations, and the connection probably takes place through a vesicular mechanism of endosomes and exosomes, as already observed in cell and rat models [31,32,33].

**Figure 1:**
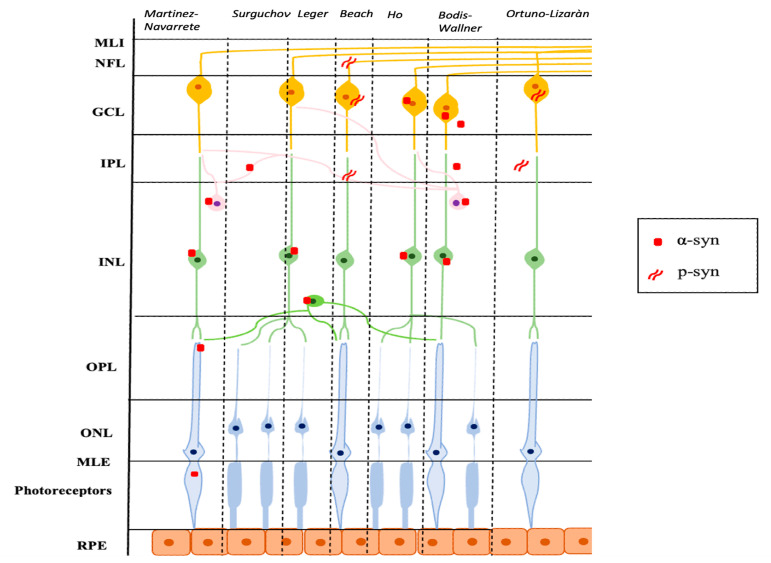
Schematic representation of the main localization of α-syn and p-syn in retinal tissue described in several studies. α-syn: α-synuclein; p-syn: phosphorylated α-synuclein; ILM: internal limiting membrane; NFL: nerve fiber layer; GCL: ganglion cell layer; IPL: inner plexiform layer; INL: inner nuclear layer; OPL: outer plexiform layer; ONL: outer nuclear layer; ELM: external limiting membrane; RPE: retinal pigment epithelium.

Ortuno-Lizaràn et al. successively carried out an investigation, similar to the research by Beach et al., with the specific objective of characterizing the cells and structures where the modified p-syn proteins accumulate and to assess a possible correlation between the amount of p-syn in the retina and the brain. They found an accumulation of p-syn in the form of axonal fibers, dendrites, and/or neuronal perikaryal structures in the retinas of patients with PD and in 3 subjects with incidental Lewy Body disease. All cells involved had morphological changes and had their cell bodies in the GCL and most of their dendrites in the IPL. In addition, the authors observed a positive correlation between the density of p-syn in the retina and in the brain of subjects with PD and a correlation with motor scores and the stage of the disease. Ortuno-Lizaràn et al. pointed out that these findings suggested that the progression of the disease is correlated in both tissues and that the retina can act as a biomarker of PD brain disease [34]. Figure 1 graphically summarizes the reported studies.

## 3. SD-OCT Findings

**Figure 2:**
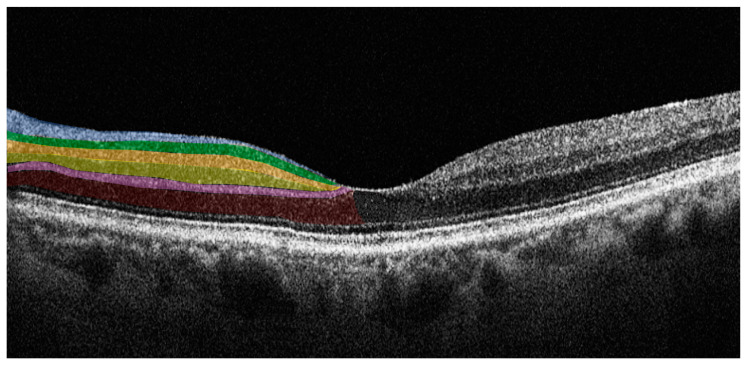
Horizontal spectral domain optical coherence tomography (SD-OCT) cross-sectional scan passing through the fovea. The various retinal layers are shown with added color overlayers on the left side as follows: blue: retinal nerve fiber layer; green: ganglion cell layer; orange: inner plexiform layer; yellow: inner nuclear layer; pink: outer plexiform layer; red: outer nuclear layer. The right side shows original SD-OCT output in scales of grey.

SD-OCT is an imaging technology that enables the evaluation of the retina in vivo, providing information on retinal morphology and thickness values of individual retinal layers with high-resolution. SD-OCT uses a near-infrared wavelength with a broad-band width light source to illuminate the retina and assess the light reflected from retinal tissue interfaces with a spectrometer and Fourier transformation. The device analyzes the reflected light creating two- and three-dimensional images with near cellular resolution (<10 μm), in which different retinal layers can be differentiated by signal intensity. The SD-OCT data-analysis program generates numerical values for retinal layer thicknesses and entire macular and foveal volumes. Furthermore, it is a non-invasive imaging technique with good sensitivity and reproducibility that is an ideal tool for the longitudinal assessment of degenerative change in the retina [35]. In PD patients there is retinal accumulation of alpha-synuclein, in its native or modified form, particularly in the inner retina. SD-OCT allows the accurate study of these layers enabling evaluation of changes in structure or thickness related to the pathology [36]. Figure 2 illustrates the individual retinal layers on a SD-OCT cross-sectional scan.

The studies in the literature that describe SD-OCT alterations in PD are numerous and have produced inconsistent results (Table 1).

Aydin et al. using SD-OCT found a reduction of the mean RNFL and retinal thickness in the nasal sector in patients with PD [37]. Similar results were found by Segupta et al., who found that peripapillary RNFL was thinner in PD patients and macular volumes were diminished in both para- and perifoveal regions but not in the central foveal area. The authors suggested that this could be due to the anatomical constitution of the fovea, which is characterized almost exclusively by the presence of photoreceptors in the center and by a more complex cellular network including ganglion cells in the periphery. Ganglion cells are the most affected cell population in early forms of PD in relation to the dopaminergic depletion of amacrine cells. This would cause a loss of retinal ganglion cells and their axons, leading to the thinning of the RNFL layer [38]. Similar results were provided by several other studies [39,40,41,42,43,56]. Rascunà et al. found a significant reduction of the macular and peripapillary RNFL and GCL, and also significantly lower IPL, INL, OPL, and ONL thickness in PD patients with respect to healthy controls [44]. These results were subsequently confirmed by these authors in another study where patients with an early form of PD and patients with idiopathic rapid eye movement sleep behavior disorder (iRBD), considered a prodromic form of PD, were included. A thinning of the different retinal layers was shown in both PD and iRBD patients compared to healthy controls, with iRBD group values that were intermediate between PD patients and controls. The authors hypothesized that retinal impairment already occurs in the prodromic phase of PD which represents an early sign of neurodegeneration. Retinal thinning may then worsen with the progression of neurodegeneration, reflecting a continuum of neuronal damage that already begins in iRBD patients and continues with the onset of PD [45]. This continuum of damage was also observed in a later study by Wang et al. in a larger cohort of patients in different stages of disease. Peripapillary RNFL, GCL, IPL and ONL, as well as total macular retinal thickness and macular volume were measured. The authors found that patients with PD had a significantly lower thickness of all parameters examined with respect to healthy controls. Interestingly, the GCL, IPL, and ONL were thinner in patients with PD with Hoehn-Yahr I (H-Y I) stage and significantly decreased as the H-Y stage increased [46]. These findings suggest that the structural damage to the retina can be contemporaneous and proportional to the progression of the disease.

Zou et al. found a reduction of the RNFL thickness only in the temporal sector in PD patients with respect to controls. Moreover, the authors found that the total macular volume, macular retinal thickness, and GCL-IPL thicknesses were reduced in eyes with PD. In line with previous authors, Zou et al. hypothesized that GCL-IPL thinning is derived from ganglion cell apoptosis secondary to dopaminergic deprivation and that the temporal reduction of RNFL thickness is related to the anatomical distribution of nerve fibers, physiologically more abundant in that area. In addition, nerve fibers and ganglion cells are absent in the fovea, thus the central macular thickness was similar between PD patients and controls, while the total macular volume and macular retinal thickness, that analyze a wider region, were clearly thinner [47].

Unlu et al. similar to the studies described, found significant thinning of the macular RNFL, GCL, IPL, ONL, and RPE in PD demonstrating the involvement of both the inner and the outer retina, consistent with the loss of retinal dopaminergic amacrine cells. Nevertheless, surprisingly, they found an increase of OPL thickness in PD patients. In reference to a previous study by Chorostecki et al. the authors hypothesized that this finding could be explained by the accumulation of α-syn within the OPL [48,57]. In a more recent study, Cesareo et al. also found an increase in OPL thickness and thinning of the ONL. However, unlike the previous results, these authors found RPE thickening in patients with PD and suggested that this was due to the accumulation of α-syn in retinal tissue. In fact, as already explained, the intraretinal accumulation of α-syn, in addition to its steric encumbrance, causes alteration of the physiological intra- and extra-cellular metabolic pathways, causing an increase in the number of oxidized proteins and toxic intracellular aggregates. Among these, ferritinophagy is one of the most affected pathways, with deleterious consequences on photoreceptors, rich in ferritinoportine and iron. Indeed, in physiological conditions, ferritinophagy induces the release of iron from ferritin in the RPE cells. The alteration of ferritinophagy due to α-syn results in an intracellular accumulation of iron [58]. This would lead to thickening of the RPE and to the reduction of ONL thickness, which contains the nuclei of photoreceptors in involution due to the direct and indirect cytotoxic effect of α-syn [49].

Several studies found no differences in SD-OCT parameters in PD patients with respect to healthy controls [50,51,52]. Gunes et al. and Robbins et al. did not find differences in RNFL and GCC thickness. Robbins et al. suggested that although α-syn is present in the retina in individuals with PD, the changes caused may be too limited to be observed with currently available instrumentation [50,52]. Bayram et al., in a study conducted on early-stage patients with AD (8 patients) and PD (13 patients) did not find significant changes in RNFL thickness, except for higher values in the superior RNFL thickness in PD patients. However, the diagnosis of patients was not confirmed by any laboratory tests or post-mortem examinations [53]. Similarly, Matlach et al., found no differences in inner retinal and average RNFL thickness between patients with PD compared to age-matched subjects, but there was a statistically significant thinning of the superior RNFL in the ipsilateral eye to the most affected side of the body with bradykinesia, suggesting laterality between brain damage and retinal damage [54]. Batum et al. in a recent study confirmed that RNFL thickness was similar in PD patients with respect to controls; however, they found a thinning of retinal thicknesses and GCC thickness in all areas. and speculated that this may be due to the short disease duration in the PD patients they studied [55].

## 4. Discussion

There is evidence in the literature that the accumulation of α-syn and, more specifically, post-translationally modified forms of α-syn, occurs in the retina as well as in the brain tissue. What remains to be understood is whether these alterations are linked to the typical visual changes in PD patients. A recent report by Marrocco et al., performed on transgenic mice, showed how overexpression of α-syn leads to neurodegeneration of tyrosine hydroxylase amacrine cells followed by a degeneration of ganglion cells with a direct consequence on visual functions such as adaptation to light and visual acuity [19]. Clearly, further in vivo studies in humans are needed to better characterize and understand the mechanisms by which these pathological accumulations of α-syn affect the dopaminergic function of visual impulse transmission.

There is a need to find a non-invasive biomarker that would facilitate diagnosis of PD, possibly years before the onset of the classic symptoms. As in other neurodegenerative diseases, the eye and in particular the retina, due to its easy accessibility and the direct derivation from neuronal tissue, could offer an early diagnosis of PD. However, there are still criticisms. The totality of available studies in the literature does not allow to define the prevalence and specificity of retinal α-syn inclusions in PD owing to the the low numbers of patients and differences in study protocols. Furthermore, future research would benefit by studies with patients in different stages of PD, possibly correlating the amount of α-syn with the occurrence of PD symptoms.

A critical point is the current possibility of observing the retina in vivo with imaging techniques in ophthalmology; research could be aimed to detect α-syn deposits in vivo, possibly differentiating modified and unmodified protein forms. Widely available imaging techniques used in PD patients are SD-OCT and confocal scanning laser ophthalmoscopy (cSLO); similar but improved technologies are needed to visualize retinal α-syn and/or p-syn and/or LBs in vivo. SD-OCT alterations in PD patients are controversial. Huang et al., in a recent meta-analysis of 27 SD-OCT studies analyzing changes in retinal thickness in PD patients versus healthy controls concluded that there is a thinning of NFL, GCL, and IPL that may be due to the dopaminergic depletion that occurs during PD, which leads to a reduced interaction between dopaminergic amacrine cells and retinal ganglion cells leading to atrophy of the ganglion cells and their nerve fibers [42]. The studies reported in this review on SD-OCT parameters in PD patients also had conflicting results. The reasons for these inconsistencies are possibly due to the methodological differences adopted such as variability in SD-OCT devices used, sample size, stage of disease of enrolled patients, and the presence of visual and systemic symptoms. Most of the studies in the literature were conducted on patients with an already advanced stage of the disease. However, few research groups detected early-stage aggregates (oligomers) or monomer accumulation before the advanced clinical manifestations [34,49,53].

Further studies are warranted in order to better characterize the retinal architectural changes in PD evidenced by SD-OCT and to possibly correlate these with the histological aspects (presence of α-syn), and with neuro-functional features (visual acuity, contrast sensitivity, intraretinal pulse transmission). For now, what we see are the indirect effects of what happens at the microscopic level; the next frontier will be to directly visualize intraretinal accumulations in vivo even before they begin to induce retinal damage. In this regard, several technologies are developing, but are unfortunately still at a level of experimentation [59,60]. One hypothesis could be the use of cSLO, a non-invasive imaging technique that allows to obtain high resolution en face images of the retina and can be used to detect fluorescence signals. This technique could be supplemented by the use of fluorescent probes capable of binding to the aggregating α-syn monomers and oligomers. Unfortunately, this new experimental technology has not yet been translated to the retina and is still a long way from clinical development [61,62].

In conclusion, numerous studies, conducted ex vivo, have shown the accumulation of α-syn and p-syn in different retinal localizations in ageing and in PD with different distribution patterns. These observations have a huge potential to lay the foundations for a better understanding of the mechanisms through which visual impairment occurs in PD and to develop an easily accessible and non-invasive biomarker for the early detection of PD, even years before the onset of symptoms. For this to happen, however, retinal imaging techniques that allow to directly observe the effects of α-syn intraretinal accumulation in vivo are required. Current available SD-OCT data is still not enough for this purpose. However, the early diagnosis and follow-up of PD may be possible with multicentric, prospective and longitudinal clinical studies on imaging techniques to better clarify the actual extent and temporal course of retinal damage in PD.

## Figures and Tables

**Table 1:** Clinical characteristics included spectral-domain optical coherence tomography studies.

Study	Number of Eyes	Parameters Measured	SD-OCT Model	Results on PD Patients	Other Findings
Aydin et al. 2018 [37]	Cases: 25 PD Controls: 29	RNFL and retinal thickness Correlation between RNFL thickness and severity of disease	Heidelberg Spectralis SD-OCT (software version 5.3; Heidelberg Engineering, Heidelberg, Germany)	Nasal retinal thickness and mean RNFL thickness reduced in PD	
Sengupta et al. 2018 [38]	Cases: 34 PD Controls: 50	RNFL thickness, macular volume	Heidelberg Spectralis HRA + OCT (Heidelberg Engineering, Heidelberg, Germany)	Reduction of RNFL thickness in PD. Macular volumes diminished in both perifoveal and outer macular regions in all sectors in PD	No differences in central foveal volume
Moschos et al. 2018 [39]	Cases: 31 PD Controls: 25	GCC, RNFL and choroidal thickness	Heidelberg Spectralis HRA + OCT (Heidelberg Engineering, Heidelberg, Germany)	Reduced GCC and RNFL thickness in PD	
Verghesee et al. 2022 [40]	Cases: 15 PD Controls: 11	Macular GCC and RNFL, peripapillary RNFL thickness	Heidelberg Spectralis HRA + OCT (Heidelberg Engineering, Heidelberg, Germany)	Reduced macular thickness, macular GCC and RNFL, and peripapillary RNFL in PD	
Huang et al. 2018 [41]	Cases: 53 PD Controls: 41	RNFL, central and mean macular thickness, and macular volumes	Zeiss Cirrus HD-4000 SD-OCT (Carl Zeiss Meditec, Inc., Dublin, CA, USA)	Macular retinal thickness, macular volume, and average RNFL thickness thinner in PD	
Huang et al. 2020 [42]	Cases: 53 PD Controls: 41	MRT, TMV and peripapillary RNFL thickness	Zeiss Cirrus HD-4000 SD-OCT (Carl Zeiss Meditec, Inc., Dublin, CA, USA)	Reduction of MRT, TMV and peripapillary RNFL in patients with advanced stage of PD versus controls	
Lee et al. 2019 [43]	Cases: 36 PD Controls: 57	Retinal, RNFL, GCL and IPL thickness	Heidelberg Spectralis SD-OCT (Heidelberg Engineering, Heidelberg, Germany)	Reduction of retinal, GCL and IPL thickness in temporal and inferior sectors. reduction of RNFL in inferior sector in PD	
Rascunà et al. 2020 [44]	Cases: 21 PD Controls: 17	Peripapillary and macular RNFL, GCL thickness, IPL thickness, and INL thickness	Zeiss Cirrus HD-5000 SD-OCT (Carl Zeiss Meditec, Inc., Dublin, CA, USA)	Peripapillary and macular RNFL, GCL, IPL, and INL thinner in PD versus HCs.	
Rascunà et al. 2021 [45]	Cases: 21 PD, 19 MSA Controls: 33	RNFL, GCL, IPL, INL, OPL and ONLthickness (both PD and iRBD patients)	Zeiss Cirrus HD-5000 SD-OCT (Carl Zeiss Meditec, Inc., Dublin, CA, USA)	RNFL, GCL, IPL, INL, OPL and ONL thinner in PD and iRBD versus controls	
Wang et al. 2022 [46]	Cases: 397 PD Controls: 427	Peripapillary RNFL thickness, MRT, and TMV	Zeiss Cirrus HD-5000 SD-OCT (Carl Zeiss Meditec, Inc., Dublin, CA, USA)	Peripapillary RNFL thickness, MRT and TMV reduced in PD	GCL, IPL and ONL thinner in patients with advanced stage of disease
Zou et al. 2020 [47]	Cases: 35 PD Controls: 35	CMT, MRT, TMV, GCL-IPL thickness, RNFL thickness	Zeiss Cirrus HD-5000 SD-OCT (Carl Zeiss Meditec, Inc., Dublin, CA, USA)	Temporal RNFL thinner in PD	No difference of mean RNFL thickness and CMT.
Unlu et al. 2018 [48]	Cases: 58 PD Controls: 30	Peripapillary and macular RNFL, GCL thickness, IPL thickness, INL, ONL, OPL, PR, RPE thickness	Heidelberg Spectralis SD-OCT (Heidelberg Engineering, Heidelberg, Germany)	Peripapillary and macular RNFL, GCL thickness, IPL thickness, INL, ONL, PR, and RPE thickness volumes lower in PD. OPL volumes increased in PD	
Cesareo et al. 2021 [49]	Cases: 41 PD Controls: 41	Peripapillary RNFL and retinal thickness	Heidelberg Spectralis SD-OCT (Heidelberg Engineering, Heidelberg, Germany)	No differences in peripapillary RNFL thickness. ONL thinner whereas OPL and RPE thicker in PD	
Günes et al. 2019 [50]	Cases: 22 PD Controls: 22	RNFL and GCC thickness	RTVue-XR Avanti (Optovue Inc., Fremont, CA, USA)	No differences in RNFL and GCC thickness	
Alkabie et al. 2020 [51]	Cases: 12 PD, 11 PSP Controls: 12	RNFL thickness and macular volume	Heidelberg Spectralis SD-OCT (software version 5.1.2; Heidelberg Engineering, Heidelberg, Germany)	RNFL thickness and macular volume not significantly different between eyes of PD patients and controls	
Robbins et al. 2021 [52]	Cases: 69 PD Controls: 137	RNFL, GCL-IPL thickness, CST, SFCT, Angio-OCT parameters, and CVI	Zeiss Cirrus HD-5000 SD-OCT (Carl Zeiss Meditec, Inc., Dublin, CA, USA)	No difference in CST, RNFL, and GCIPL thickness between PD and controls.	Increased total choroidal and choroidal luminal area; decreased CVI in PD
Bayram et al. 2019 [53]	Cases: 15 PD, 15 AD Controls: 15	RNFL, macular, foveal and parafoveal thickness	Rtvue OCT (Optivue INC, Toredo, OH, USA)	Superior RNFL and superior retinal thickness higher in PD	Temporal, inferior and inferior retinal and RNFL thinner in PD (not statistically significant).
Matlach et al. 2018 [54]	Cases: 30 PD Controls: 40	GCC and peripapillary RNFL thickness	Zeiss Cirrus HD-4000 SD-OCT (Carl Zeiss Meditec, Inc., Dublin, CA, USA)	No differences in RNFL and GCC thickness	Superior RNFL significantly thinner in ipsilateral eye (not contralateral eye) to the most-affected body side with bradykinesia of PD patients compared to controls
			RTVue-100^®^ (Optovue Inc., Fremont, CA, USA; software version 6.3)		
Batum et al. 2022 [55]	Cases: 50 PD, 15 MSA Controls: 50	GCC and peripapillary RNFL thickness	Stratus OCT (Carl Zeiss Meditec Inc., Dublin, CA, USA)	Retinal thickness and GCC thinner in PD versus HCs. No significant difference in peripapillary RNFL thickness in all quadrants between PD and controls	

PD: Parkinson’s disease; PSP: progressive supranuclear palsy; OCT: optical coherence tomography; RNFL: retinal nerve fiber layer; GCL-IPL: ganglion cell–inner plexiform layer; CSF: central subfield thickness; SFCT: subfoveal choroidal thickness; CVI: choroidal vascularity index; CMT: central macular thickness; MRT: macular retinal thickness; TMV: total macular volume; GCC: ganglion cell complex ; IPL: inner plexiform layer; ONL: outer nuclear layer; OPL: outer plexiform layer; MSA: multi systemic atrophy; iRBD: idiopathic rapid-eye-movement sleep behavior disorder; ET: essential tremor; RPE: retinal pigment epithelium.

## Data Availability

Not applicable.
